# The role of testosterone in male sexual function

**DOI:** 10.1007/s11154-022-09748-3

**Published:** 2022-08-23

**Authors:** Giovanni Corona, Mario Maggi

**Affiliations:** 1 Endocrinology Unit, Medical Department, Maggiore-Bellaria Hospital, Azienda Usl, Bologna, Italy; 2 Endocrinology Unit, Department of Experimental and Clinical Biomedical Sciences, University of Florence, Florence, Mario Serio Italy

**Keywords:** Testosterone, Hypogonadism, Testosterone replacement therapy, Erectile dysfunction

## Abstract

Sexual function, and testosterone (T) levels, progressively decline in aging men. Associated morbidities and metabolic disorders can accelerate the phenomenon. The specific contribution of low T to sexual function impairment in aging men has still not been completely clarified. Similarly, the role of T replacement therapy (TRT), as well as the combination of TRT with phosphodiesterase type 5 inhibitors (PDE5i) for patients with erectile dysfunction (ED), is still conflicting. Here we aim to summarize and critically discuss all available data supporting the contribution of low T to sexual impairment observed with aging as well as the possible role of TRT. Available data on men with sexual dysfunction show that reduced sexual desire is the most important correlate of male hypogonadism. Conversely, aging and associated morbidities substantially attenuate the relationship between ED and T. TRT is effective in improving sexual function in middle-aged and older subjects but its role is small and extremely variable. Lifestyle interventions can result in similar outcomes to those of TRT. In conclusion, it is our opinion that PDE5i along with lifestyle measures should be considered the first approach for treating ED even in subjects with milder T deficiency. When these interventions fail or are difficult to apply, TRT should be considered.

## Introduction

Successful aging has been considered one of the main aspects of all societies since the Roman Empire. In the *“Cato Maior de senectute*”, Marcus Tullius Cicero, when he was 62 years old, concluded that the cornerstone for successful aging should be based on a combination of both adequate physical and mental lifestyle behaviors “…*to adopt a regimen of health, to practice moderate exercise, to take just enough food and drink, to restore our strength and not to overburden it. Nor, indeed are we to give our attention solely to the body. Much greater care is due to the mind and soul”.* In line with Cicero’s thoughts, the progressive improvement of economic, social, cultural and medical conditions, which have occurred particularly during the last two centuries, has allowed a tremendous global rise in life expectancy at birth from less than 30 years, at the beginning of last century, to over 72 years in 2019 (https://ourworldindata.org/life-expectancy). The progressive increase of the proportion of older people represents also a source of new challenges for all health-care professionals, policy advisers and decision-making organizations in order to guarantee social and medical support to permit an active and adequate quality of life for this aging population [1].

According to the World Health Organization, sexual health is fundamental to the overall health and well-being of individuals, couples and families, and to the social and economic development of communities and countries (https://www.who.int/health-topics/sexual-health#tab=tab_1). Several population-based studies have documented that, despite an age-dependent decline of sexual function, a large proportion of older adults are still interested in sexual activities, with men being more frequently sexually active compared to women [2–6]. The same studies have clearly demonstrated that, according to psychological, organic and couple relationship modifications, occurring with advanced age, coital intercourse is not an essential prerequisite to remain sexually active [7, 8]. In line with the latter finding, erectile dysfunction (ED) concern declines with aging [4, 5, 7, 9].

Despite this evidence, plenty of data has recognized that ED should be considered an early marker of forthcoming cardiovascular (CV) mortality and morbidity [10]. Accordingly, ED shares several traditional risk factors with CV diseases (CVD) [10, 11]. Hence, available data clearly indicate that sexual health should be considered a mirror of general health, which, in turn, is a prerequisite to remaining sexually active. In line with these considerations, the concept “sexually active life expectancy” defined as the “the average number of years remaining spent as sexually active” has been introduced [12]. According to epidemiological data, men present a potentially longer sexually active life expectancy when compared to women; however, the real period of a sexually active life is reduced due to poorer general health [12].

Human sexuality is the result of a complex interaction between the endocrine milieu, general health, psychological well-being and couple health [13–19]. The perturbation of any domain in one of the partners has detrimental effects on the couple, eventually leading to an overall marital and sexual health impairment [20–22]. Interestingly, we previously reported that, among subjects seeking medical care for ED, not only organic but also relational and intrapsychic factors can contribute to the stratification of CV risk [23]. In older people, lifestyle changes occurring with advancing age – including: the death of their partner, worsening of social status, deterioration of support networks and health and finance-related family problems—might contribute to sexual difficulties. This change, by inducing and contributing to the development of depressive and anxiety symptoms as well as couple health perturbation, can, in turn, worsen and aggravate the CV risk profile [23].

Testosterone (T) is a well-recognized crucial factor in regulating male sexual response acting either at a central or peripheral level [17, 18, 24]. Several studies performed in community dwelling men have documented that aging is associated with the progressive decline of T circulating levels [25–31]. Late onset hypogonadism (LOH) is the most frequently used term to describe the latter phenomenon [32]. Although the specific mechanisms underlying LOH have not been completely clarified, mounting evidence has pointed out that associated morbidities, and in particular metabolic disorders, can play a critical role [33, 34]. However, it is important to recognize that LOH per se has been associated with worse metabolic and CV profiles [35, 36] although data derived from interventional studies are still conflicting [37, 38]. The European Male Aging Study (EMAS), a population-based survey performed on more than 3400 men recruited from eight European centers, clearly showed that sexual symptoms—particularly ED, decreased frequency of morning erections and of sexual thoughts—are the most sensitive and specific symptoms in identifying patients with LOH [39]. Similar results were recently reported by our group in a large cohort (n = 4890) of subjects consulting for ED [40]. In contrast, psychological and physical symptoms were less informative [39].

The aim of the present study is to summarize and critically discuss all available data supporting the role of T on the regulation of erectile function in aging men. Other aspects of sexual function including libido and ejaculation will also be analyzed. In addition, the possible contribution of T replacement therapy (TRT) to sexual outcomes as well as the role of the combined therapy with other ED drugs such as phosphodiesterase type 5 inhibitors (PDE5i) will also be addressed.

## Methods

A comprehensive narrative review was performed using Medline, Embase and Cochrane search and including the following words: ("testosterone"[MeSH Terms] OR "testosterone"[All Fields] OR "testosteron"[All Fields] OR "testosterones"[All Fields] OR "testosterone s"[All Fields]) AND ("sexual behavior"[MeSH Terms] OR ("sexual"[All Fields] AND "behavior"[All Fields]) OR "sexual behavior"[All Fields] OR "sexual"[All Fields] OR "sexually"[All Fields] OR "sexualities"[All Fields] OR "sexuality"[MeSH Terms] OR "sexuality"[All Fields] OR "sexualization"[All Fields] OR "sexualize"[All Fields] OR "sexualized"[All Fields] OR "sexualizing"[All Fields] OR "sexuals"[All Fields]) AND ("functional"[All Fields] OR "functional s"[All Fields] OR "functionalities"[All Fields] OR "functionality"[All Fields] OR "functionalization"[All Fields] OR "functionalizations"[All Fields] OR "functionalize"[All Fields] OR "functionalized"[All Fields] OR "functionalizes"[All Fields] OR "functionalizing"[All Fields] OR "functionally"[All Fields] OR "functionals"[All Fields] OR "functioned"[All Fields] OR "functioning"[All Fields] OR "functionings"[All Fields] OR "functions"[All Fields] OR "physiology"[MeSH Subheading] OR "physiology"[All Fields] OR "function"[All Fields] OR "physiology"[MeSH Terms]) AND ((male[Filter]) AND (English [Filter])). Publications from January 1, 1969 up to December 31, 2021 were included.

Clinical data were derived from a consecutive series of 3500 patients seeking medical care at the University of Florence as previously described (see also Table 1 [16]).

**Table 1:** Relationship between several sexual symptoms and quintiles of age, total testosterone (T) and chronic disease score (CDS, a broad index of associated morbidities) as derived from multivariate binary logistic model to explain moderate or severe sexual symptoms. Data are adjusted for the other two variables

**Loss of erection (n = 3621)**			
	Wald	Exp(B) [95% CI]	Significance (p)
Total T (quintiles)	8.2	0.889[0.821;0.964]	0.004
Age (quintiles)	60.292	1.467[1.332;1.616]	<0.0001
CDS (quintiles)	17.508	1.231[1.117;1.357]	<0.0001
**Hypoactive sexual desire (n = 3624)**			
	Wald	Exp(B) [95% CI]	Significance (p)
Total T (quintiles)	26.989	0.841[0.788;0.898]	<0.0001
Age (quintiles)	10.614	1.129[1.049;1.214]	0.001
CDS (quintiles)	2.467	0.937[0.863;1.016]	0.116
**Loss of spontaneous erection (n = 3601)**			
	Wald	Exp(B) [95% CI]	Significance (p)
Total T (quintiles)	8.699	0.927[0.882;0.975]	<0.0001
Age (quintiles)	104,436	1.343[1.269;1.421]	<0.0001
CDS (quintiles)	62.819	1.287[1.209;1.370]	<0.0001
**Frequency of sexual intercourse (n = 3410)**			
	Wald	Exp(B) [95% CI]	Significance (p)
Total T (quintiles)	15.071	0.906[0.862;0.952]	<0.0001
Age (quintiles)	39.841	1.196[1.132;1.265]	<0.0001
CDS (quintiles)	15.462	1.136[1.066;1.211]	<0.0001

## Aspect of male sexual function most closely related to androgen activation

Male sexual function is an absolute prerequisite for species perpetuation, because it allows semen deposition within the female vagina, and, thereafter, eventual oocyte fertilization. However, in humans, normal sexual functioning is also an important part of couple relationships, allowing for the exchange of intimacy and pleasure between two partners.

Normal male sexual function is usually categorized into four potentially progressive steps: libido, erection, orgasm with ejaculation and, eventually, detumescence [41]. The male gonadal hormone T is potentially involved in regulating all these steps. Low T is, in fact, often associated with reduced libido, ED, reduced spontaneous erection and delayed ejaculation. The first three associations have been observed not only in the general population [39] but also in hypogonadal subjects (e.g. Klinefelter’s Syndrome or subjects undergoing androgen deprivation therapy, [42–45] as well as in subjects complaining for sexual dysfunction (see in [46] for review)). In contrast, the relationship between delayed ejaculation and T deficiency is more debated [19, 47].

**Fig. 1:**
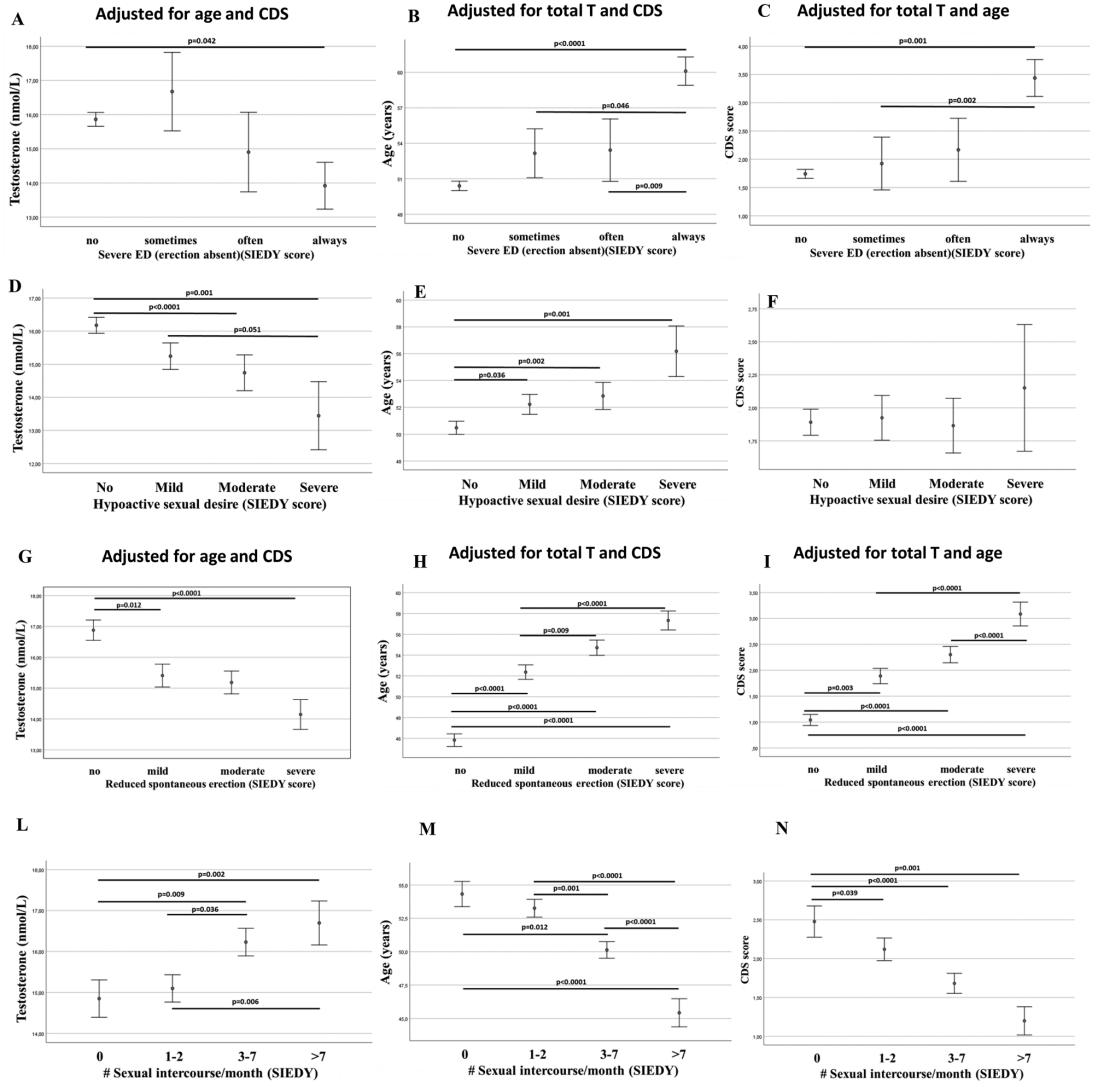
Relationships among T levels (**A**, **D**, **G**, **L**), age (**B**, **E**, **H**, **M**), comorbidities (as detected by Chronic Disease Score; CDS; **C**, **F**, **I**, **N**) and several symptoms of an impaired male sexuality; in subjects consulting for sexual dysfunction at the university of Florence, Florence, Italy (see also Table 1). ED = erectile dysfunction; SIEDY = Structured Interview on Erectile Dysfunction. Reported significances are those derived after adjusting each association for the other two determinants using ANOVA and post-hoc Bonferroni test

Although the association between T deficiency and sexual dysfunction is statistically significant in different populations [39, 42–45], its specificity is rather low, because other determinants may cause, or at least be concurrent in explaining, the sexual symptoms. For instance, male sexual dysfunctions always have a dyadic declination; in fact, an impaired couple relationship as well as conflicts within the couple—or even within the family—could be associated with a T decline [48, 49]. In addition, the aging process *per se* or the presence of associated morbidities could often underlie impaired sexual functioning. Figure 1 shows the relationships among T levels, age, comorbidities and several symptoms of an impaired male sexuality, as referred by subjects complaining for sexual dysfunction in a public andrology center in Italy. Data are derived from published series concerning almost 3500 patients [16, 50]. The left columns show (Fig. 1, Panels A,D,G,L) the association between symptom severity and T levels, the middle columns (Fig. 1, Panels B,E,H,M) show associations with age, and the right columns (Fig. 1, Panels C,F,I,N) association with morbidities as assessed by Chronic Disease Score (CDS), an aggregate comorbidity measure based on current medication use [16]. Reported significances are those derived after adjusting each association for the other two determinants. As shown, each reported symptom could be explained either by age or morbidities as well as by T deficiency, although to a different extent. In particular, lower T levels showed the weakest association with severe ED, which is better explained by an older age or by a higher morbidity index (Fig. 1, upper panels). Low sexual desire is the most genuine symptom associated with low T, because it can also be explained by low T and by an older age, but not by the presence of comorbidities. To confirm this point we simultaneously introduced quintiles of the three variables (age, T and CDS) in a binary logistic model in order to explain moderate or severe sexual symptoms (Table 1). After adjusting for the other two variables, the highest likelihood ratio (Wald test) for low T is with low sexual desire, while loss of erection (either spontaneous or sexual related) is better explained by an older age or by high CDS scoring. Although a relatively high likelihood ratio was observed for an impaired sexual desire with low T without the contribution of medical comorbidities (Table 1), it should be recognized that low sexual desire is often associated with several other determinants, including relational and intrapsychic ones, which play a role at least as important as the T deficiency [51].

According to the Diagnostic and Statistical Manual of Mental Disorders (DSM)-5, male hypoactive sexual desire disorder (MHSDD) is a long-lasting condition causing clinically significant distress and not accounted for by another psychiatric disorder and must not be due exclusively to the physiological effects of a substance or a general medical condition [52]. Considering that T deficiency is a general medical condition, it cannot be ascribed as a primary cause of MHSDD [53]. Similar considerations should be applied to other hormonal disorders (hyperprolactinemia) or to psychiatric conditions, such as depression. We, therefore, previously considered the latter as secondary causes of MHSDD [53]. In a survey conducted almost 10 years ago in an andrological setting for sexual dysfunctions [53], we found that only 40% of men with low T (< 12 nmol/L) show secondary MHSDD, while in those with hyperprolactinemia the symptom was almost universally present (85%). Subjects with low T and reduced sexual desire were characterized by an impaired couple relationship, higher education, and an increased level of anxiety and depressive symptoms. In addition, they showed more often severe ED, reduced spontaneous erection and reduced frequency of intercourse [53].

For all the aforementioned considerations, we believe that reduced sexual desire is the most important correlate of male hypogonadism. It can substantially impair couple relationship, mood and sexual habits. Although low sexual desire can have other determinants and is not universally present in men with low T, we believe that checking T levels in those complaining of reduced libido is an important medical action, because low desire is, in this case, a treatable condition. In fact, in all the meta-analyses of intervention trials published so far, treating low T is associated with improved sexual desire [46]. However, it is still a matter of debate whether or not the effect of T on sexual desire is due to the activation of the androgen receptor (AR) present in several areas of the human brain. In a randomized clinical trial (RCT) of daily transdermal administration of dihydrotestosterone (DHT, a non-aromatizable AR super-agonist) to 114 healthy, older men for 24 months it was found that sexual desire not only did not increase but even decreased [54] upon treatment. The latter effect was reversible after cessation of treatment [54]. In another noteworthy study by Finkelstein et al. [55], 400 healthy men were given monthly injections of goserelin, a gonadotropin-releasing hormone (GnRH) analogue, to suppress endogenous T for four months. Men were then randomly assigned to a placebo or increasing doses of transdermal T gel with or without an aromatase inhibitor (anastrozole) to suppress the conversion of T to estradiol. The study showed that both estrogen and T deficiencies contributed to the decrease in sexual functioning. In particular, in the T treated group inhibition of estrogen synthesis was associated with a significant decrease in sexual desire [55]. Thus, the local production of estrogen centrally, by means of aromatization of T, is crucial for the stimulation of sexual desire in men. However, it is important to recognize that commonly available immunoassays cannot provide sufficiently reliable estimation of estrogen circulating levels in men [24]. Hence, the latter results cannot be used during routine daily practice.

## Relationships between erectile dysfunction and T deficiency

As extensively reviewed elsewhere [46, 56, 57], most of the signaling pathways controlling penile erection are androgen-dependent, including nitric oxide (NO) production and degradation, adenosine signaling, calcium sensitization through the RhoA-ROCK pathway and even penile smooth muscle differentiation. Nonetheless, in men complaining for sexual dysfunction, self-reported severe ED and loss of spontaneous erections are only weakly associated with low T (see Fig. 1, Panels A and G, respectively and Table 1). In contrast, increasing age and level of comorbidities largely explain the variance of the sample (Table 1).

**Fig. 2:**
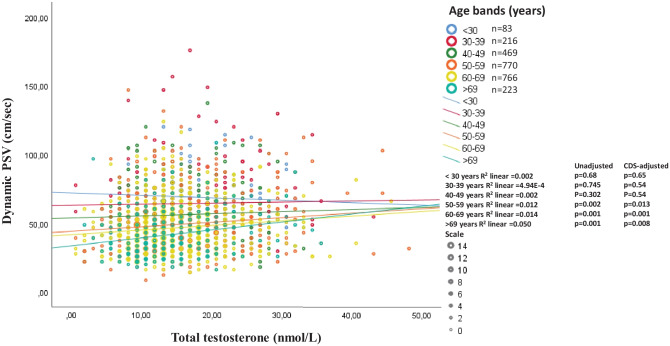
Unadjusted and (age and chronic disease score, CDS) adjusted relationships (derived from fitting regression model) between prostaglandin E1 (PGE1)-stimulated penile blood (dynamic peak systolic velocity, PSV) at penile doppler ultrasound and total testosterone in a cohort of more than 2500 men complaining of sexual dysfunction at the university of Florence, Florence, Italy

Penile erection is a neuro-vascular phenomenon in which the final step is an increase in blood flow within the lacunar spaces of the penis. To objectively evaluate a possible association between endogenous T levels and penile blood flow, we reanalyzed [58, 59] the relationship between prostaglandin E1 (PGE1)-stimulated penile blood flow increase and T levels in a cohort of more than 2500 men complaining of sexual dysfunction. In an age- and CDS-adjusted multiple regression model, T levels were positively associated with an increased penile blood flow (β = 0.069 p = 0.001), but, again, age and CDS explain the majority of the variance (β = -0.249, p < 0.0001 and β = -0.203, p < 0.0001). When the relationship was analyzed according to age bands (decades), in both an unadjusted as well as in a CDS-adjusted fitting regression, it retained significance only in the oldest (> 50 years) age bands (Fig. 2). This is tantamount to say that the modest contribution of circulating T levels to a PGE1-induced increase in penile blood flow is apparent only in middle-aged and older subjects. Interestingly, as shown in Fig. 2, the age bands over 50 years represent the large majority of subjects consulting for sexual dysfunction, at least in our Unit. In a previous analysis of the same data, we demonstrated that the effect of endogenous T on penile blood flow was apparent only in the lowest quintile of T levels, i.e. lower than 10.4 nmol/L, but less evident in higher quintiles. Hence, the positive relationship between circulating T and penile blood flow might have, if any, clinical significance in those with a low T level and an older age.

## Effect of testosterone replacement therapy in men with erectile dysfunction

**Fig. 3:**
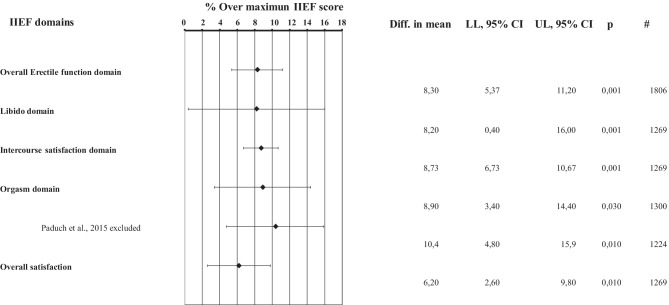
Percentage of efficacy against placebo after normalization for the maximal effect in the different International Index of Erectile Function subdomains as derived from the most recent meta-analysis on testosterone replacement therapy in sexual function [59]

Considering that association does not mean causation, only intervention studies could shed light on whether or not T administration might improve erectile function. Five meta-analyses are available analyzing the effect of TRT on sexual function, including ED, and critically analyzed elsewhere [46]. When only subjects with low T (10 nmol/L in three and 12 nmol/L in two studies) were considered, erectile function improved in all the meta-analyses with a standardized mean difference ranging from 0.16 [0.06; 0.27] to 1.87 [0.31; 3.43] [46]. It is important to note that randomized controlled trials (RCTs) included in the aforementioned meta-analyses not only differ in their definition of low T, but also in the type and dose of TRT, in the length of the studies and, more importantly, in the instruments employed to investigate erectile function. When only studies employing the universally recognized instrument for scoring ED—i.e. the International Index of Erectile Function (IIEF, [60])—were selected, the TRT-induced improvement in IIEF-erectile function domain (EFD) resulted as being relatively modest: mean difference = 2.31 [1.41; 3.22] points of IIEF-EFD [61]. Even considering the most recent trials published, the result did not substantially change [59]. In fact, the improvement in IIEF-EFD score was 2.49 [1.61–3.36] points [59]. It should be considered that the effect of the different PDE5i resulted in a 6–7 point improvement in IIEF-EFD [62] with a percentage of efficacy against placebo ranging from 20–50%. [63, 64]. Figure 3 shows the percentage of efficacy against placebo after normalization for the maximal effect in the different IIEF subdomains as derived from the most recent meta-analysis on TRT in sexual function [59]. TRT induced a modest 8% increase in IIEF-EFD. The modest increase in IIEF-EFD score induced by TRT should be considered clinically meaningful only in patients with milder forms of ED according to Rosen et al. [65]. It is important to note that in the majority of trials included in the meta-analysis [59], middle-aged and older men were enrolled, those in which, according to Fig. 2, TRT should induce the maximal increase in penile blood flow.

In Fig. 3 the effect of TRT on other IIEF subdomains is also reported, as a percentage of the maximal improvement over placebo. The percentage of improvement is always small, below 10%, even in the sexual desire domain that shows the largest confidence interval (8.2 [0.4;16]). Overall, these data indicate that TRT is effective in improving sexual function in middle-aged and older subjects with variable definitions of low T (from lower than 8 to lower than 15 nmol/L), but the effect is small and highly variable.

## Proportion of men presenting with ED and T deficiency and possible determinants

In a previous study [66], concerning endocrine abnormalities occurring in the general population of Florence—n = 202 men aged 40–79 years, a spinoff of the EMAS study ([67]: EMAS cohort) – and in a population of the same age consulting for sexual dysfunction at our Unit (UNIFI cohort n = 3847), we found that hypogonadism (total T < 10.5) was enriched in those complaining for sexual symptoms. In particular, secondary (luteinizing hormone, LH ≤ 9.4 U/L) and primary (LH > 9.4 U/L) hypogonadism were present in 8% vs. 18.9% and in 0% vs. 2.8% in the EMAS and UNIFI cohort, respectively. The difference in the prevalence of secondary hypogonadism between the two populations was significant in an age-adjusted model [66]. This suggests that more than 20% of middle-aged and older men complaining for sexual dysfunction has low T; in addition, the largest proportion of those showed normal-inadequately low LH (secondary hypogonadism). A similar figure was reported in a larger cohort (n = 4220) of men consulting for sexual dysfunction at any age [68]. Overall, 3.2% and 17.4% of enrolled men showed primary and secondary hypogonadism, respectively. Interestingly, when enrolled subjects were categorized according to the nature of the disorder, only 50% and 10% of primary and secondary hypogonadism, respectively, showed a definitive diagnosis (i.e. organic hypogonadism, [69]), whereas the rest of the sample was defined as “unknown” (i.e. functional hypogonadism, [69]). Interestingly, more than 70% of functional primary and secondary hypogonadism showed metabolic abnormalities, including diabetes mellitus, metabolic syndrome and obesity [69]. It is important to note that the latter conditions, i.e. central obesity and type 2 diabetes mellitus (T2DM), were more often present in the aforementioned UNIFI cohort consulting for sexual dysfunction (31.7% and 20.3%, respectively) than in the general Florence population (EMAS cohort 22.8% and 1%, respectively) [66]. In a more recent analysis [70], we reported that the prevalence of low T (< 10.5 nmol/L) among a consecutive series of 4412 subjects seeking medical care for sexual dysfunction was 40.7% in patients with obesity (Body mass Index, BMI > 30 kg/m^2^), 30.7 in those with T2DM and 35% in those with metabolic syndrome (MetS). Similar data were observed when low calculated free T (cFT < 225 pmol/L) was considered: 43.4%, 37.7%, and 37.9% for obesity, T2DM and MetS, respectively. Again, in subjects with metabolic conditions, secondary hypogonadism was at least five-times more prevalent than the primary form [70]. Overall, the aforementioned data indicate that secondary (central) hypogonadism of functional origin is the prominent form of T deficiency in subjects with sexual dysfunction and that the large majority of cases are associated with metabolic conditions. Although this is true for the majority of cases, still 10% of those with secondary hypogonadism showed any form of abnormality at the hypothalamic and/or pituitary level [66]. Magnetic resonance imaging (MRI) could ideally help in diagnosing organic forms of secondary hypogonadism, but it is an expensive procedure, not widely available and still burdened by long waiting lists. We recently found [71] that in both an exploratory sample (n = 126) and in the whole validation sample (n = 50) of men consulting for sexual dysfunction with secondary hypogonadism, total T ≤ 6.1 nmol/L or LH ≤ 1.9 U/L should raise the suspicion of hypothalamus/pituitary structural abnormalities, warranting an MRI evaluation (odd ratio for pituitary abnormalities = 5.49 [1.32;22.85], and 3.31 [1.02;10.72] for total T and LH, respectively).

Considering the close association among T deficiency, sexual function and metabolic impairment [39], the working mechanisms underlying the metabolic-induced impairment in the hypothalamus-pituitary-testis axis and their consequences on penile erection will be better analyzed. We previously set up an animal model of MetS, feeding rabbits for three months with a westernized diet enriched in cholesterol [72]. These rabbits developed the full human phenotype of MetS along with erectile dysfunction and secondary hypogonadism [72]. In fact, within the preoptic area of the hypothalamus we observed a MetS-induced low-grade inflammation and discrete alterations in the complex network of neurotransmitters controlling the production and secretion of GnRH [72, 73]. In addition, metaflammation –a tissue inflammation driven by metabolic disorders- was also observed within the testis [74] and epididymis [75] with a consequent impairment in steroidogenesis and spermatogenesis. Interestingly, training the MetS-rabbits to perform daily physical exercise (PhyEx) on a treadmill normalized gonadotropins and T levels and restored acetylcholine (Ach)-induced relaxation of corpora cavernosa and NO signaling within the penis [74]. The effect of PhyEx on Ach-induced relaxation was comparable to that of treating MetS with metformin or treating T deficiency with exogenous T [70]. In addition, within the hypothalamus, PhyEx reverted the majority of MetS-induced alterations in GnRH-controlling neurons and normalized preoptic metaflammation [74]. Within the testis, PhyEX increased the expression of genes related to androgen formation, as 3β hydroxysteroid dehydrogenase and its related androstenedione/testosterone ratio, and normalized macrophage infiltration (35).

Hence, based on these preclinical studies [72–75], lifestyle measures, such as performing regular PhyEx, should be considered as the optimal treatment for men with metabolic disturbances, low T and ED, i.e. for at least 20% of subjects consulting for sexual dysfunction. Is this true in clinical practice?

In a recent meta-analysis of the available trials on the effect of dieting or performing PhyEx on T levels [34] we reported that these lifestyle measures were associated with an overall 2–3 nmol/l increase in T levels. For low calorie diet, the effect was proportional to the degree of weight loss, reaching a 7 nmol/L increase in total T for a delta weight of 25 kg. A similar increase was observed in all five meta-analyses published so far on the effect of bariatric surgery on T levels, as reviewed elsewhere [34]. In particular, bariatric surgery was able to revert the condition of secondary hypogonadism associated with massive obesity, by increasing gonadotropin levels and decreasing estrogen levels [34]. For PhyEx, the effect on total T was more apparent in older individuals, in those that lose more weight and in those that exercised for a longer time [34]. Accordingly, in another meta-analysis [76], the effect of an acute PhyEx on T levels was addressed. A statistically significant effect was found, that was, however, less clinically relevant (0.7 nmol/L). Hence, prolonged lifestyle intervention appears ideal because it can not only increase endogenous T, but also ameliorate the underlying metabolic disturbances, such as obesity, MetS and even T2DM. However, effects of these lifestyle strategies in improving sexual life are less defined. In a meta-analysis on 38 population-based studies [11], it was reported that regular PhyEx was associated with a 50% reduction of the risk of ED, with the effect of prolonged PhyEx (e.g., 30 min of moderate- or high-intensity activity 5 times per week) more evident than that of a moderate activity (e.g., 20 min of moderate-intensity activity 3 times per week). In the same meta-analysis it was found that also a healthy diet, such as the Mediterranean one, is associated with a reduced risk of ED (-14%), although the effect was no longer significant after adjusting for publication bias [11]. Seven RCTs investigated the effect of intervention with aerobic, pelvic or combined PhyEx on ED, as measured by IIEF-EFD [77]. Surprisingly, the meta-analytic effect was similar to that of a weaker PDE5i, such as avanafil [64], with an increase of almost 4 points of IIEF-EFD [77]. The effect of bariatric surgery was recently investigated in two separate meta-analyses, both showing a significant improvement in erectile function (IIEF) upon treatment [78, 79], which was estimated as a 4-point increase in IIEF-EFD [79]. These increases are almost double that obtained with TRT in the general population with low T [59]. In addition, a meta-regression analysis of RCTs [59] suggests that the positive effect of TRT on erection is considerably attenuated according to the proportion of obese men enrolled in trials. It should be considered that in subjects with complicated metabolic conditions, such as an established T2DM, the effect of TRT was almost null [70]. However, a recently published Australian trial—enrolling 1007 men aged 50 to 74 years (20% with T2DM) who had a waist circumference ≥ 95 cm along with impaired glucose tolerance or newly diagnosed T2DM aimed at determining whether TRT was able to prevent transition from prediabetes to diabetes—found that those in the TRT arm had a significant (p = 0.0004) 2.10 (0.95; 3.26) point increase in IIEF-EFD over the placebo [80]. Interestingly, both arms underwent an intensive lifestyle intervention [80]. IIEF-EFD increase in the TRT arm was rather similar to that of previous RCTs, as derived from meta-analyses [46, 61]. All the other IIEF subdomains, including libido, showed improvement similar or even superior to those of previous studies [46, 61]. This means that in those with prediabetes, or with a newly diagnosed T2DM, TRT is able to improve sexual symptoms. In contrast, in RCTs enrolling subjects with an established diabetes the effect of TRT is not apparent [70], probably because it is buried by the diabetes-associated angiopathy and neuropathy.

## Screening men with sexual dysfunction for T deficiency

Although all the major guidelines suggest against a universal screening of T deficiency, the latter is recommended only in those that are persistently symptomatic for this deficiency [24, 81–83]. Considering that sexual dysfunctions are universally considered a correlate of T deficiency [24, 81–83], it is obvious that in men persistently complaining for sexual dysfunctions T levels should be adequately checked, i.e. in the early morning after adequate fasting [24, 81–83]. Calculation of the proportion of free T, by sex hormone binding globulin (SHBG) measuring, along with measurement of LH level, to determine the nature of the T failure, is also recommended [24, 81–83]. Hence, in our opinion, there is no doubt that T deficiency should be adequately investigated in all men presenting with sexual dysfunction.

## Testosterone added on to PDE5i

**Fig. 4:**
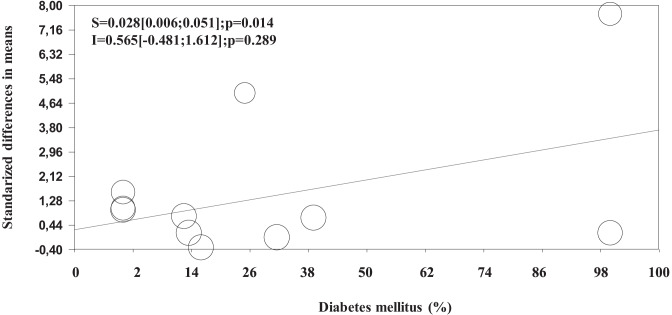
Influence of baseline diabetes mellitus prevalence on erectile function of combined therapy (testosterone and phosphodiesterase type 5 inhibitors, PDE5i) versus PDE5i alone. Results are derived from the analysis of previously reported data [85]

Considering the relatively modest effect of TRT on erectile function, several studies have attempted to evaluate whether the combined therapy of TRT and PDE5i can result in better outcomes [38, 50, 84, 85]. In a pioneering prospective randomized placebo-controlled study, Aversa et al. [86] reported, for the first time, that a combined therapy between transdermal T and sildenafil resulted in better IIEF score improvement when compared to sildenafil and placebo. The same study also documented that the combined therapy significantly improved penile arterial flow as detected by penile color Doppler ultrasound [86]. The latter finding can partly explain the observed results, since the possible role of TRT in improving endothelial function has been reported by other authors [87–89], although the topic is still controversial [90]. The specific mechanisms supporting these effects are reviewed elsewhere [89]. In addition, as previously reported, the effects of T on several pathways controlling penile erection is also well documented [17]. By combining all the available evidence in a first meta-analysis published in 2014—including 12 studies [86, 91–101], enrolling 894 patients with a mean follow-up of 12 weeks—we documented [85] that the combined therapy between TRT and PDE5i was associated with better outcomes, when compared to controls, only when non placebo-controlled RCT were considered (see also Tables 2 and 3). Conversely, no significant effects were observed when placebo-controlled RCT were evaluated (see also Table 3) [85]. In a more recent meta-analysis, including 8 placebo-controlled RCT—encompassing 913 patients with a mean follow-up of 10.8 weeks—Zhu et al. [84] concluded that the combined therapy was superior when compared to PDE5i alone in improving erectile function. The same study also documented better outcomes when only trials including hypogonadal patients (total T < 10 nM) were considered [84]. However, it should be recognized that the latter study presents important limitations. In particular, 4 out of 8 included trials were articles published only in the Chinese language and not available in PubMed. Hence, the latter results should be interpreted with caution, due to the possible presence of selection biases. After our previous meta-analysis [85], no further placebo-controlled RCT available in PubMed addressing this topic was published. Interestingly, by reconsidering all the available studies (including uncontrolled ones) from our previous meta-analysis [85], we originally report here that the combined therapy resulted in significantly better outcomes, particularly when those trials including patients not responding to PDE5i alone at enrolment were considered (Table 3). In addition, meta-regression analysis of those data shows that the combined effects were significantly higher in those trials including a larger amount of diabetic patients (Fig. 4). Since the effects of PDE5i [102] or TRT alone [70] in subjects with diabetes are limited, the present results suggest that a combination therapy should be suggested in more complicated subjects or at least in those with diabetes mellitus. However, it should be recognized that when only studies using IIEF-EFD as outcomes were considered, no significant advantage in either placebo-controlled or not controlled studies was observed (Table 3). In considering the effects of TRT in placebo-controlled RCT it should be recognized that 2 [86, 98] out of 4 of the included trials enrolled a mixed population of eugonadal and hypogonadal men. In addition, although Spitzer et al.’s trial [99] included only hypogonadal subjects at enrolment, TRT was initiated after a sildenafil alone run-in period which resulted in T increase up to the normal range (about 12.0 nmol/L) [103]. Hence, although preliminary results suggest that the combined use of TRT and PDE5i could result in better outcomes in more complicated patients, more studies are advisable to draw final conclusions.

**Table 2:** Characteristics and outcomes of the controlled clinical studies included in the meta-analysis

**Study (ref.)**	**# patients (T + PDE5i/** **PDE5i ± ** **placebo)**	**Placebo controlled**	**PDE5i non responders**	**Trial duration (weeks)**	**Age (years)**	**T levels**	**Diabetes (%)**	**Dose** **(daily)**	**Design**
Aversa et al. [86]	10/10	+	+	4	48–66	Mixed	25	T patch 50 mg/day	Parallel
Kalinchenko et al. [91]	120/120	-	+	4	43–74	<8 nM	100	TU 160 mg/day	BA
Foresta et al. [92]	15/15	-	-	24	32.1 ± 2.6	<8 nM	0	TG 50 mg/day	BA
Shabsigh et al. [93]	39/36	-	+	12	18–80	Mixed	16	TG 50 mg/day	Parallel
Shamloul et al. [94]	10/10	-	+	8	40–70	<8 nM	0	TU 120 mg/day	Parallel
Hwang et al. [95]	32/21	-	+	12	23–73	12 nM	12.5	TU 160–240 mg/day	BA
Rochira et al. [96]	24/24	-	-	24	35.0 ± 12.0	<8 nM	0	TG 50 mg/day	Cross-Over
Garcia et al. [97]	29/29	-	+	102	36–75	<12 nM	NR	TU1000mg/12 weeks	BA
Buvat et al. [98]	87/86	+	+	12	45–80	Mixed	32	TG 50 mg/day	Parallel
Spitzer et al. [99]	70/70	+	-	14	40–70	<12 nM	13.5	TG100 mg/day	Parallel
Kim et al. [100]	44/34	-	+	24	41–75	<12 nM	39	TE 250 mg/month	BA
Hackett et al. [101]	14/14	+	+	30	33–83	<12 nM	100	TU1000mg/12 weeks	Parallel

All data are reported as mean ± SD

*BA* Controlled cohort before-and-after comparisons, *TE* testosterone enanthate, *TU* testosterone undecanoate, *TC* testosterone cypionate, *NR* not reported. Adapted from ref. [85]

**Table 3:** Effect size (with 95%CI) of testosterone replacement therapy as add on to phosphodiesterase type 5 inhibitors (PDE5ì) in placebo controlled or uncontrolled trials (RCT; see also Table 1)

	**Outcomes**
**Overall population** *Non placebo controlled RCT (n* = *7)* *Placebo controlled RCT (n* = *5)*	SMD 2.96[1.28;4.64]; p < 0.0001 0.49[-0.16;1.15]; p = 0.140
**Overall population** PDE5i non responders *(n* = *8)* *General population (n* = *4)*	SMD 2.77[1.35;4.20]; p < 0.0001* 0.63[0.04;1.22]; p = 0.04
**Only EFD score** *Non placebo controlled RCT (n* = *3)* *Placebo controlled RCT (n* = *4)*	Mean difference in EFD 8.94[-3.27;21.15]; p = 0.151 2.88[-1.89;7.64];p = 0.237

Data are expressed in the overall population or when only those trials enrolling PDE5i non responders or International Index of erectile function –erectile function domain score (IIEF-EFD) was used as outcomes

*Q = 7.45; p = 0.01 vs general population. Results are derived from data included in ref [85]

## Conclusions

Sexual dysfunctions, such as ED and MHSDD, are associated with low T levels. This point is well accepted by all the major guidelines on the topic [24, 81–83]. Considering that loss of libido is less affected by age-associated comorbidities, it represents the most genuine symptom of T deficiency in adulthood. In contrast, other predictors, such as age and comorbidities, more often determine ED. The negative contribution of low T to an impaired penile blood flow is more apparent in older than in younger subjects. The most important correlates of low T in subjects with sexual dysfunction are metabolic disturbances, including obesity, MetS and T2DM, most probably because they are associated with metaflammation of hypothalamic centers known to regulate GnRH production and release. Accordingly, secondary hypogonadism is by far the most prevalent form of T deficiency in adulthood. Gaining weight, and in particular abdominal obesity, is an important predictor of a forthcoming secondary hypogonadism [104]. Weight reduction by lifestyle measures is associated with an increase in circulating gonadotropins and T levels, along with an improvement in erectile function. Meta-analyses showed here and elsewhere [59, 61] indicate that TRT has a marginal effect (less than 10%) in restoring sexual life in otherwise hypogonadal subjects. Such an effect appears even lower than that obtained with lifestyle measure, such as physical activity, although the number of RCTs available is scanty [11, 77]. In addition, the positive effect of TRT on ED is less apparent in subjects with established metabolic disturbances, such as T2DM [70]. PDE5i are three times more effective in treating ED than TRT [62]. Although PDE5i, such as tadalafil, might be less effective in men with low T (< 10.4 nmol/L), one trial still indicated an increase of 6 points IIEF-EFD [105]. Available data published so far cannot clarify the real significance of the combined therapy of T and PDE5i, although preliminary results suggest a possible role in more complicated patients. Larger placebo-controlled RCT are advisable to better clarify this topic. Hence, a PDE5i along with lifestyle measures—such as moderate physical activity, smoking cessation and dieting—are the most appropriate interventions for treating ED even in subjects with T deficiency. It should be considered that these lifestyle measures not only improve sexual life but also treat underlying metabolic conditions, often present in these subjects. When these interventions fail or are not applicable, TRT should be considered. TRT shows the advantage of improving body composition [70], including muscle mass, and, possibly, ameliorating glucose control [80], even in subjects with metabolic conditions. In addition, it can have a positive effect on sexual desire, whereas all the PDE5i are almost ineffective and no other specific treatment is available.

## Abbreviations

AR: Androgen receptor
BMI: Body mass index
CDS: Chronic Disease Score
CV: Cardiovascular
CVD: Cardiovascular diseases
DHT: Dihydrotestosterone
DSM-5: Diagnostic and Statistical Manual of Mental Disorder 5th Edition.
ED: Erectile dysfunction
EFD: Erectile function domain
EMAS: European Male Aging Study
GnRH: Gonadotropin-releasing hormone
IIEF: International Index of Erectile Function
LH: Luteinizing hormone
LOH: Late onset hypogonadism
MRI: Magnetic resonance imaging
MHSDD: Male hypoactive sexual desire disorder.
MetS: Metabolic syndrome
NO: Nitric oxide
PCDU: Penile color Doppler ultrasound
PDE5i: Phosphodiesterase type 5 inhibitor
PGE1: Prostaglandin E1
PhyEx: Physical exercise
RCT: Randomized controlled trials
SIEDY: Structured Interview on Erectile Dysfunction
T: Testosterone
T2DM: Type 2 diabetes mellitus
TRT: Testosterone replacement therapy
